# Systemic immune-inflammation index is associated with clinical outcome of acute ischemic stroke patients after intravenous thrombolysis treatment

**DOI:** 10.1371/journal.pone.0319920

**Published:** 2025-03-27

**Authors:** Yuanfeng Zhou, Qian Yang, Zhangming Zhou, Xin Yang, Danni Zheng, Zhongchun He, Yizhou Liu, Tianzhu Xu, Ying Yin, Wenhui Wei, Chunli Si, Bozhi Zhang, Jianping Yu

**Affiliations:** 1 Chengdu Medical College, Chengdu, Sichuan, China; 2 Department of Neurology, The First Affiliated Hospital of Chengdu Medical College, Chengdu, Sichuan, China; 3 Department of Neurosurgery, Dujiangyan Medical Center, Chengdu, Sichuan, China; 4 Biomedical Informatics and Digital Health, School of Medical Sciences, University of Sydney, Sydney, Australia; 5 Department of Medical Administration, The First Affiliated Hospital of Chengdu Medical College, Chengdu, Sichuan, China; West China Hospital of Sichuan University, China

## Abstract

**Introduction:**

The systemic immune-inflammation index (SII) has been proven to predict the outcome in cancerous and non-cancerous diseases. We aimed to investigate the relationship between SII and other inflammatory markers and the prognosis in patients receiving intravenous thrombolysis (IVT).

**Methods:**

Acute ischemic stroke patients treated with IVT were collected retrospectively. SII, neutrophil-to-lymphocyte ratio (NLR) and platelet-to-lymphocyte ratio (PLR) were constructed based on admission blood testing. Favorable outcome was defined as modified Rankin Scale of less than or equal to 2 at 90 days. In addition to outcome, cerebral edema was analyzed. The severity of brain edema was graded into three levels according to Thrombolysis in Stroke-Monitoring Study. Malignant cerebral edema (MCE) was defined as brain edema with midline shift.

**Results:**

278 patients were included. 140 (50.4%) achieved favorable outcome, 35 (12.6%) developed MCE. In patients with favorable outcomes, the levels of SII, NLR and PLR were lower compared to those with unfavorable outcomes [422.33 (258.69-624.68) vs 1269.83 (750.82-2497.22), p < 0.001; 2.73 (1.68-4.40) vs 4.76 (2.59-7.72), p < 0.001; 92.98 (62.35-126.24) vs 115.64 (85.51-179.04), p < 0.001]. The area under the Receiver Operating Characteristic curve was 0.698 for SII (95% CI = 0.637-0.760, p < 0.001), 0.694 for NLR (95% CI = 0.632-0.756, p < 0.001), 0.643 for PLR (95% CI = 0.579-0.707, p < 0.001). The optimal cut-off values were 652.73 for SII (sensitivity 0.572, specificity 0.786), 3.57 for NLR (sensitivity 0.659, specificity 0.693), 127.01 for PLR (sensitivity 0.457, specificity 0.757).

**Conclusions:**

An early increase in SII levels was related to 3 months of unfavorable outcomes in AIS patients after IVT. However, it is not associated with malignant edema.

## Introduction

Stroke was the third leading cause of death and the fourth leading cause of Disability-Adjusted Life Years (DALYs) globally and it was also the most significant health burden across all regions in East Asia in 2021 [1]. Acute ischemic stroke (AIS) constitutes 70% of all stroke cases. Intravenous thrombolysis (IVT) is the first line treatment recommended by most guidelines [2,3]. However, IVT for AIS patients remains underused, only 5.64% eligible AIS patients receiving IVT in China [4]. One of the common reasons for non-treatment was the concern about the development of severe complications after reperfusion therapies. Spontaneous intracerebral hemorrhage (sICH) and malignant cerebral edema (MCE) are regarded as devastating complications of IVT, with incidence rates of 10.3% and 28%, respectively [5–7]. Nowadays, the predictors for the development of MCE and sICH for AIS patients after IVT were uncertain.

Inflammation is a critical pathological process of ischemic stroke and severe AIS complications like sICH and MCE. Infiltrating blood-borne immune cells (neutrophils, monocytes, and T lymphocytes) increase blood-brain barrier permeability and exacerbated brain edema by inducing microvascular disruption and secreting inflammation-related molecules [8]. Some inflammatory factors, such as neutrophil counts, neutrophil-to-lymphocyte ratio (NLR), lymphocyte-to-monocyte ratio, platelet-to-lymphocyte ratio (PLR), platelet-to-neutrophil (PNR), neutrophil/ (leucocyte- neutrophil) and red cell volume distribution width were reported to be related to clinical outcomes in AIS patients [9–11]. Systemic immune-inflammatory index (SII) is a novel inflammatory indicator, which plays an important role in predicting the prognosis and survival rate of patients with brain malignant tumor, spontaneous cerebral hemorrhage, aneurysmal subarachnoid hemorrhage, cerebral infarction and dementia [11–15]. Increased baseline SII was associated with 3-month unfavorable outcomes in AIS patients who underwent IVT [16–18].

AIS patients who received IVT and developed malignant edema were more likely to experience unfavorable outcomes. However, the relationship between the SII at admission and malignant edema in thrombolyzed patients remains unclear. We hypothesized that elevated SII is associated with an increased risk of malignant brain edema after IVT, potentially leading to poorer outcomes. Our objectives were to (1) examine the association between early SII and 3-month clinical outcomes in patients who received only IVT, and (2) explore the association between early SII and malignant brain edema in these patients.

## Methods

### Participants

We retrospectively identified thrombolytic AIS patients who were admitted to the First Affiliated Hospital of Chengdu Medical College from January 2018 to December 2021. The inclusion criteria were: 1) had a clinical diagnosis of AIS, confirmed by brain computed tomography (CT) or magnetic resonance imaging (MRI); 2) underwent IVT; and 3) had complete laboratory and clinical data and imaging scans. The exclusion criteria included: 1) missed laboratory data or loss to follow-up; 2) had a history of malignant tumors; 3) had severe chronic liver or kidney diseases; 4) had hematologic disorders, active infectious diseases or received immunosuppressive treatment; 5) received endovascular treatment (EVT).

This study was conducted in accordance with the declaration of Helsinki. We obtained ethical approval for this study from the Medical and Health Research Ethics Committee of the Chengdu Medical College, under approval number 2023CYFYIRB-BA-Jun07. Data were accessed for this research purposes at 2023.7.5. Due to the retrospective observational nature of this study and the blinding of subject names, the ethics committee waived the requirement for informed consent.

### Data collection

We retrieved demographic and clinical data. The results of the total blood count were used to calculate biochemical indicators of SII, NLR and PLR [19,20]. $\text{SII}=\left[\text{neutrophil count*platelet count}\right]∕\text{lymphocyte count}$. NLR=neutrophil count∕lymphocyte count. PLR=platelet count∕lymphocyte count. Demographic data, vascular risk factors, medical history, and baseline stroke severity according to the National Institutes of Health Stroke Scale (NIHSS) score were retrieved from medical records. The NIHSS score difference was defined as the NIHSS score at admission minus the NIHSS score at 24 hours after admission. The ischemic lesion extension was estimated according to the Alberta Stroke Program Early CT Score (ASPECTS) on head non contrast CT performed at the Emergency Department.

A head CT scan or magnetic resonance imaging was performed 24 hours after IVT for assessment of cerebral edema (CED). The severity of the CED was further assessed by the two radiologists independently grading from the Safe Implementation of Thrombolysis in Stroke-Monitoring Study (SITS-MOST) [21]. Of these, brain edema was classified into three levels, CED-1 (focal brain edema up to one-third of the hemisphere), CED-2 (brain edema greater than one-third of the hemisphere), and CED-3 (brain edema with midline shift, MLS) [22]. CED-3 was defined as MCE.

The functional outcome measured using the modified Rankin Scale (mRS) score was ascertained at follow-up at outpatient clinic or through telephone interview three months after the onset of symptoms. The primary outcomes include unfavorable clinical outcome, defined as having a mRS score greater than or equal to 3 at 90 days. The second outcome includes the severity of brain edema.

### Statistical analysis

All the statistical analysis was performed using SPSS (version 25.0, IBM Corp, Armonk, NY, USA). Continuous variables were presented as mean ±  standard deviation (SD, normal distribution) or median and inter-quartile range (IQR, non-normal distribution). Categorical variables were expressed as percentages.

All patients were dichotomized according to the 3-month mRS scores (favorable 0-2 vs. unfavorable 3-6). Student t test or Mann Whitney U test for continuous variables. The χ^2^ test was used for categorical variables. The variables with p-values < 0.05 from the comparison of baseline characteristics were considered confounding factors and were entered into the binary logistic regression model (stepwise analysis). Given that SII, NLR, and PLR are calculated using platelet counts, neutrophil counts, and lymphocyte counts, these variables often exhibit relatively small variations. To improve the data distribution and amplify the effect sizes, we log-transformed SII, NLR, and PLR. The transformed values were then re-analyzed using binary logistic regression. The optimal cut-off values for predicting the prognosis of AIS patients who underwent IVT were evaluated by receiver operating characteristic curve (ROC). A two-tailed p-value of < 0.05 was considered statistically significant.

## Results

### Baseline characteristics

**Fig 1:**
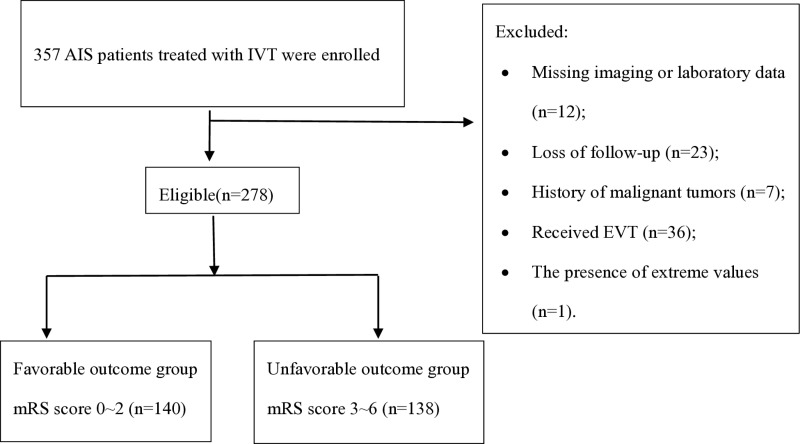
Patients selection flowchart. Study design with inclusion and exclusion criteria for AIS patients who underwent thrombolysis. From these 357 patients, 278 (77.9%) patients met the criteria. AIS, acute ischemic stroke; IVT, intravenous thrombolysis; mRS score, the modified Rankin Scale score; EVT, endovascular treatment.

A total of 357 patients who received IVT were reviewed, and 278 eligible patients were included (Fig 1). 12 patients were excluded for missing imaging or laboratory data. 23 patients were loss of follow-up, 7 patients were excluded for malignant tumors; 36 patients were excluded for bridging treatments after IVT and 1 patient was excluded for the presence of extreme values. The median age was 74 years (range, 23-92 years), 135 (48.6%) patients were women. The median baseline NIHSS score was 10 (inter-quartile range, 5-15). The median serum SII level was 508.74 (inter-quartile range, 321.76-963.23). The median serum PLR level was 104.37 (inter-quartile range, 73.48-153.42). The median serum NLR level was 3.49 (inter-quartile range, 2.08-5.80). A total of 138 (49.6%) patients experienced unfavorable outcome. Follow-up scans conducted 24 hours after treatment revealed malignant brain edema in 35 patients (12.6%).

### Association between SII, NLR, PLR and clinical unfavorable outcome

According to the mRS score at 3 months after symptom onset, patients were divided into favorable outcome (mRS score 0-2, n = 140, 50.4%) and unfavorable outcome (mRS score 3-6, n = 138, 49.6%). The baseline characteristics of patients are shown in Table 1.

**Table 1:** Demographics and clinical characteristics of the subgroup according to clinical outcomes.

Variables	3 Months outcome			Brain edema	
	Favorable outcome (n = 140)	Unfavorable outcome (n = 138)	Non-malignant brain edema (n = 243)	Malignant brain edema(n = 35)	P value
**Demographics**					
Age, y; median (IQR)	71 (62–79)	77 (68–82)	**<0.001**	78 (71.83)	**0.044**
Male, sex; n (%)	78 (55.7%)	65 (47.1%)	0.187	14 (40.0%)	0.154
**Previous History n (%)**					
Hypertension, n (%)	70 (50.0%)	82 (59.4%)	0.119	22 (62.9%)	0.365
DM, n (%)	63 (45.0%)	79 (57.2%)	**0.043**	18 (51.4%)	1.000
CAD, n (%)	20 (14.3%)	13 (9.4%)	0.266	5 (14.3%)	0.583
HL, n (%)	84 (60.0%)	79 (57.2%)	0.715	18 (51.4%)	0.365
AF, n (%)	46 (32.9%)	65 (47.1%)	**0.020**	19 (54.3%)	0.068
Stroke, n (%)	12 (8.6%)	16 (11.6%)	0.432	7 (20.0%)	0.064
Smoking, n (%)	42 (30.0%)	34 (24.6%)	0.348	9 (25.7%)	1.000
Drinking, n (%)	29 (20.7%)	21 (15.2%)	0.275	6 (17.1%)	1.000
**Baseline Parameters**					
SBP (mmHg); mean ± SD	152.88 ± 22.05	159.19 ± 26.40	**0.031**	164.20 ± 25.73	**0.034**
DBP (mmHg); median (IQR)	81.00 (74.00-90.00)	81.50 (73.75-95.50)	0.624	84 (74,101)	0.379
ONT, mean ± SD	156.82 ± 53.28	171.17 ± 53.91	**0.026**	163.69 ± 51.58	0.976
ASPECTS score; median (IQR)	8 (7–9)	7 (6–8)	**<0.001**	7 (6–7)	**<0.001**
mRS score; median (IQR)	0 (0–0)	0 (0–1)	**0.010**	0 (0–1)	0.119
Initial NIHSS Score; median (IQR)	6 (4–11)	13 (9–17)	**<0.001**	16 (13–18)	**<0.001**
24h NIHSS score; median (IQR)	2 (1–6)	13 (6–18)	**<0.001**	17 (13–20)	**<0.001**
NIHSS difference; median (IQR)	2 (0-5)	0 (0–3)	**<0.001**	0 (-3-2)	**0.001**
MCE; n(%)	1 (0.7%)	34 (12.6%)	**<0.001**		
**Laboratory Data**					
Glu; median (IQR)	6.91 (5.82–8.39)	7.65 (6.45–9.88)	**0.003**	8.15 (7.04 –10.50)	**0.013**
Neutrophil(×10^9^/L); median (IQR)	4.31 (3.62–5.85)	5.82 (4.15–9.40)	**<0.001**	4.89 (3.45–9.44)	0.854
Monocyte(×10^9^/L); median (IQR)	0.51 (0.41–0.65)	e	0.263	0.49 (0.38–0.64)	0.206
Lymphocyte(×10^9^/L); median (IQR)	1.64 (1.20–2.38)	1.32 (0.89–1.90)	**<0.001**	1.44 (0.99–1.79)	0.318
HB(g/L); mean ± SD	134.34 ± 16.43	130.75 ± 17.13	0.076	131.34 ± 19.77	0.648
PLT (×10^9^/L); median (IQR)	154.50 (122.00–185.00)	163.00 (131.00–201.00)	0.115	143.00 (114.00–199.00)	0.411
PT; median (IQR)	11.00 (10.43– 11.50)	10.30 (10.70–11.80)	**0.026**	11.30 (11.00–11.90)	0.051
D-dimer (mg/L); median (IQR)	364.00 (156.50–1148.75)	863.50 (293.25–2199.75)	**<0.001**	1273.00 (539.00–3163.00)	**<0.001**
TT (s); median (IQR)	14.00 (13.10–15.00)	14.30 (13.40–15.20)	0.251	14.30 (13.70–15.20)	0.154
INR; median (IQR)	1.02 (0.96–1.07)	1.05 (0.99–1.10)	**0.019**	1.05 (1.01–1.11)	**0.046**
APTT; median (IQR)	30.60 (28.33–33.00)	30.35 (27.65–32.83)	0.242	31.00 (27.00–32.80)	0.897
FIB (g/L); mean ± SD	3.60 ± 0.81	3.95 ± 0.93	**<0.001**	4.08 ± 0.94	**0.030**
FDP; median (IQR)	4.06 (1.47–13.16)	10.19 (3.00–23.98)	**<0.001**	15.07 (3.19–38.10)	**0.003**
Cr (μmol/L); median (IQR)	68.75 (57.30–85.13)	70.05 (59.18-88.13)	0.295	73.90 (59.70-93.20)	0.266
UA (μmol/L); median (IQR)	344.00 (292.00-395.00)	340.50 (269.50-437.25)	0.950	328.00 (270.00-438.00)	0.738
Apolipoprotein AI; median (IQR)	1.28 (1.08-1.46)	1.28 (1.12-1.42)	0.995	1.31 (1.19-1.42)	0.206
Lp(a); median (IQR)	136.50 (89.00-231.88)	174.00 (92.50-287.00)	0.108	229.00 (109.00-335.00)	0.054
ALB(g/L); median (IQR)	40.20 (37.50-43.00)	40.25 (36.78-43.00)	0.506	40.50 (37.20-42.80)	0.798
Triglyceride; median (IQR)	1.27 (0.89-1.95)	1.09 (0.80-1.61)	0.158	1.15 (0.78-1.61)	0.807
CHOL (mmol/L); median (IQR)	4.35 (3.64-4.89)	4.38 (3.81-5.07)	0.385	4.30 (3.79-5.07)	0.899
LDL (mmol/L); median (IQR)	2.54 (1.91-2.95)	2.54 (1.91-3.19)	0.476	2.55 (1.93-3.41)	0.629
HbA1c(%);median (IQR)	5.95 (5.60-6.45)	6.20 (5.70-5.63)	**0.024**	6.30 (5.70-7.00)	0.051
Hcy (μmol/L); median (IQR)	14.30 (10.83-17.33)	13.85 (10.88-17.70)	0.907	14.20 (12.40-17.50)	0.283
SII; median (IQR)	422.33 (258.69-624.68)	1269.83 (750.82-2497.22)	**<0.001**	511.08 (317.31-1230.41)	0.825
NLR; median (IQR)	2.73 (1.68-4.40)	4.76 (2.59-7.72)	**<0.001**	3.60 (2.20-7.11)	0.383
PLR; median (IQR)	92.98 (62.35-126.24)	115.64 (85.51-179.04)	**<0.001**	104.52 (80.00-136.36)	0.850

DM, diabetes; CAD, coronary artery disease; HL, hyperlipidemia; AF, atrial fibrillation; SBP, systolic blood pressure, DBP, diastolic blood pressure; ONT, onset to door time; ASPECT score, alberta stroke program early CT score; mRS score, the modified Rankin Scale score; NIHSS score, the national institute of health stroke scale score; MCE, malignant cerebral edema; Glu, glucose; HB, hemoglobin; PLT, platelet; PT, prothrombin time; TT, thrombin time; INR, international normalized ratio; APTT, activated partial thromboplastin time; FIB, fibrinogen; FDP, fibrinogen degradation products; Cr, creatinine; UA, uric acid; Lp(a), lipoprotein(a); ALB, albumin; CHOL, cholesterol; LDL, low-density lipoprotein; HbA1c%, hemoglobin A1c; Hcy, homocysteine; SII, systemic immune-inflammation index; NLR, neutrophil-to-lymphocyte ratio; PLR, platelet-to-lymphocyte ratio.

Patients with favorable outcome were younger [71.00 (62.00-79.00) vs. 77.00 (68.00-82.00), p < 0.001], lower rate of diabetes[63 (45.0%) vs. 79 (57.2%), p = 0.043], lower rate of atrial fibrillation[46 (32.9%) vs. 65 (47.1%), p = 0.020], shorter time of onset-to-door time (ONT) [156.82 ± 53.28 vs. 171.17 ± 53.91, p = 0.026], lower NIHSS score on admission [6 (4–11) vs. 13 (9–17), p < 0.001], lower NIHSS score at 24 hours after admission [2 (1–6) vs. 13 (6–18), p < 0.001], higher ASPECTS score on admission [8 (7–9) vs. 7 (6–8), p < 0.001], Patients with favorable outcome had a greater functional improvement after thrombolysis compared to those with unfavorable outcome: NIHSS difference [2 (0–5) vs. 0 (0–3), p < 0.001].

In terms of laboratory findings, patients with favorable outcome had higher level of prothrombin time (PT) [11.00 (10.43-11.50) vs. 10.30 (10.70-11.80), p = 0.026], lower levels of glucose [6.91 (5.82-8.39) vs.7.65 (6.45-9.88), p = 0.003], fibrinogen (FIB) [3.60 ± 0.81 vs. 3.95 ± 0.93, p < 0.001], fibrinogen degradation products (FDP) [4.06 (1.47-13.16) vs. 10.19 (3.00-23.98), p < 0.001], hemoglobin A1c (HbA1c%) [5.95 (5.60-6.45) vs. 6.20 (5.70-5.63), p = 0.024]. Neutrophils was significantly decreased in the favorable outcome group [4.31 (3.62-5.85) vs. 5.82 (4.15-9.40), p < 0.001]. Patients with favorable outcome had a higher level of lymphocytes [1.64 (1.20-2.38) vs. 1.32 (0.89-1.90), p < 0.001]. SII, NLR and PLR levels in the favorable outcome group were significantly lower than that in the unfavorable outcome group [(422.33 (258.69-624.68) vs 1269.83 (750.82-2497.22), p < 0.001; 2.73 (1.68-4.40) vs 4.76 (2.59-7.72), p < 0.001; 92.98 (62.35-126.24) vs 115.64 (85.51-179.04), p < 0.001)].

After adjusting for potential confounders mentioned, higher levels of SII, NLR, and PLR were independently associated with unfavorable outcome (odds ratio [OR], 1.001 [95% CI, 1.001-1.002], p < 0.001; OR, 1.268 [95% CI, 1.133-1.420], p < 0.001; OR, 1.009 [95% CI, 1.003-1.014], p < 0.001) in the binary stepwise logistic regression analysis (Table 2).

**Table 2:** Associations between SII, NLR, PLR and 3-months unfavorable clinical outcome.

Variables	OR	95%CI	P value
**Model 1** ^a^			
24h NIHSS score	1.221	1.147-1.299	<0.001
ASPECTS score	0.705	0.522-0.953	0.023
Non-diabetes	0.479	0.250-0.916	0.026
Non-MCE	0.090	0.010-0.768	0.028
SII	1.001	1.001-1.002	<0.001
**Model 2** ^a^			
mRS score	1.719	1.013-2.917	0.045
24h NIHSS score	1.222	1.149-1.299	<0.001
Non-diabetes	0.416	0.215-0.802	0.009
Non-MCE	0.079	0.009-0.668	0.020
NLR	1.268	1.133-1.420	<0.001
**Model 3** ^a^			
24h NIHSS score	1.226	1.153-1.303	<0.001
ASPECTS score	0.717	0.535-0.961	0.026
Non-diabetes	0.430	0.227-0.815	0.010
Non-MCE	0.034	0.012-0.839	0.034
PLR	1.009	1.003-1.014	<0.001

^a^Adjusted for confounders including age, history of atrial fibrillation, history of diabetes, SBP, mRS score, initial NIHSS score, NIHSS score in 24hours, difference of NIHSS score, ASPECTS score, malignant, glucose, prothrombin time, fibrinogen, fibrinogen degradation products, HbA1C%. NIHSS score, the national institute of health stroke scale score; ASPECT score, alberta stroke program early CT score; MCE, malignant cerebral edema; mRS score, the modified Rankin Scale score; SII, systemic immune-inflammation index; NLR, neutrophil-to-lymphocyte ratio; PLR, platelet-to-lymphocyte ratio.

After performing log-transformation on SII, NLR, and PLR, and conducting binary stepwise logistic regression analysis again, we found that the risk of poor prognosis of AIS increased by 18.207 times for each SD increase of SII (p < 0.001), increased by 15.604 times for each SD increase of NLR (p < 0.001) and increased by 17.826 times for each SD increase of PLR (p < 0.001) (Table 3).

**Table 3:** Associations between logSII, logNLR, logPLR and 3-months unfavorable clinical outcome.

Variables	OR	95%CI	P value
**Model 1** ^a^			
Age	1.029	0.999-1.061	0.061
24h NIHSS score	1.231	1.154-1.131	<0.001
ASPECTS score	0.758	0.555-1.036	0.023
Non-diabetes	0.500	0.257-0.973	0.026
Non-MCE	0.088	0.010-0.747	0.028
logSII	18.207	5.738-57.772	<0.001
**Model 2** ^a^			
Age	1.035	1.004-1.068	0.029
mRS score	1.852	1.041-3.295	0.036
24h NIHSS score	1.253	1.169-1.342	<0.001
Non-diabetes	0.435	0.222-0.852	0.015
atrial fibrillation	2.357	1.060-5.241	0.035
Non-MCE	0.069	0.008-0.588	0.014
logNLR	15.604	4.898-49.712	<0.001
**Model 3** ^a^			
24h NIHSS score	1.234	1.159-1.313	<0.001
ASPECTS score	0.724	0.540-0.970	0.030
Non-diabetes	0.422	0.222-0.803	0.009
Non-MCE	0.104	0.012-0.889	0.039
logPLR	17.826	4.062-78.233	<0.001

^a^Adjusted for confounders including age, history of atrial fibrillation, history of diabetes, SBP, mRS score, initial NIHSS score, NIHSS score in 24hours, difference of NIHSS score, ASPECTS score, malignant, glucose, prothrombin time, fibrinogen, fibrinogen degradation products, HbA1C%. NIHSS score, the national institute of health stroke scale score; ASPECT score, alberta stroke program early CT score; MCE, malignant cerebral edema; mRS score, the modified Rankin Scale score; SII, systemic immune-inflammation index; NLR, neutrophil-to-lymphocyte ratio; PLR, platelet-to-lymphocyte ratio.

The logistic regression analysis revealed that high SII, NLR and PLR were significantly related to the poor functional prognosis of AIS.

**Fig 2:**
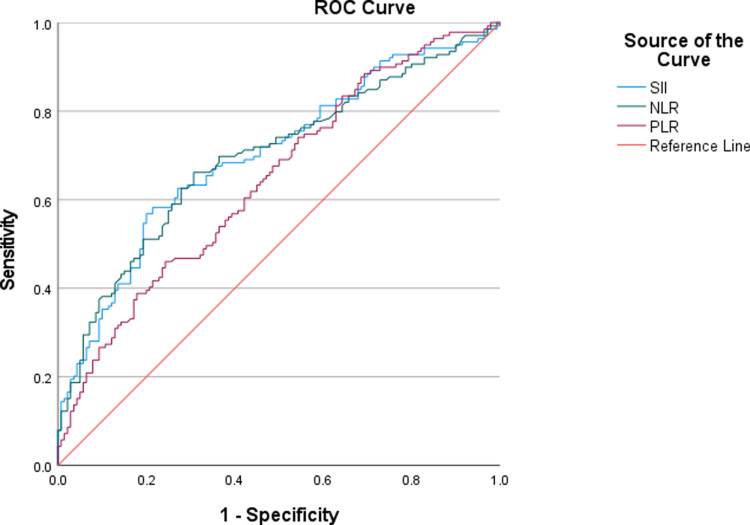
ROC analysis of SII, NLR, PLR for predicting unfavorable 3-month outcome. ROC curves of SII, NLR and PLR were analyzed to predict the clinical outcome of thrombolysis. In ROC analysis examining the association between SII, NLR and PLR values and 3-month unfavorable clinical outcome, the optimal cutoff values for SII, NLR and PLR were identified as 652.73, 3.57 and 127.01, respectively. The area under the curve (AUC) for SII was 0.698 (95% CI: 0.637–0.760, p <  0.001), for NLR was 0.694 (95% CI: 0.632–0.756, p <  0.001), and for PLR was 0.643 (95% CI: 0.579–0.707, p <  0.001). ROC, receiver operating characteristic curve; SII, systemic immune-inflammation index; NLR, neutrophil-to-lymphocyte ratio; PLR, platelet-to-lymphocyte ratio.

To explore the ability of SII, NLR and PLR to predict unfavorable clinical outcome in AIS, we performed ROC curve (Fig 2). The ROC curve showed the optimal SII cut-off value that best distinguished unfavorable outcome was 652.73 ([AUC] 0.698, 95% CI 0.637-0.760, p < 0.001), with a sensitivity of 57.2% and a specificity of 78.6%. The optimal cut-off value of NLR was3.57 ([AUC] 0.694, 95% CI 0.632-0.756, p < 0.001), with a sensitivity of 65.9% and a specificity of 69.3%. The optimal cut-off value of PLR was 127.01 ([AUC] 0.643, 95% CI 0.579-0.707, p < 0.001), ([AUC] 0.643, 95% CI 0.579-0.707, p < 0.001), with a sensitivity of 45.7% and a specificity of 75.7%.

**Fig 3:**
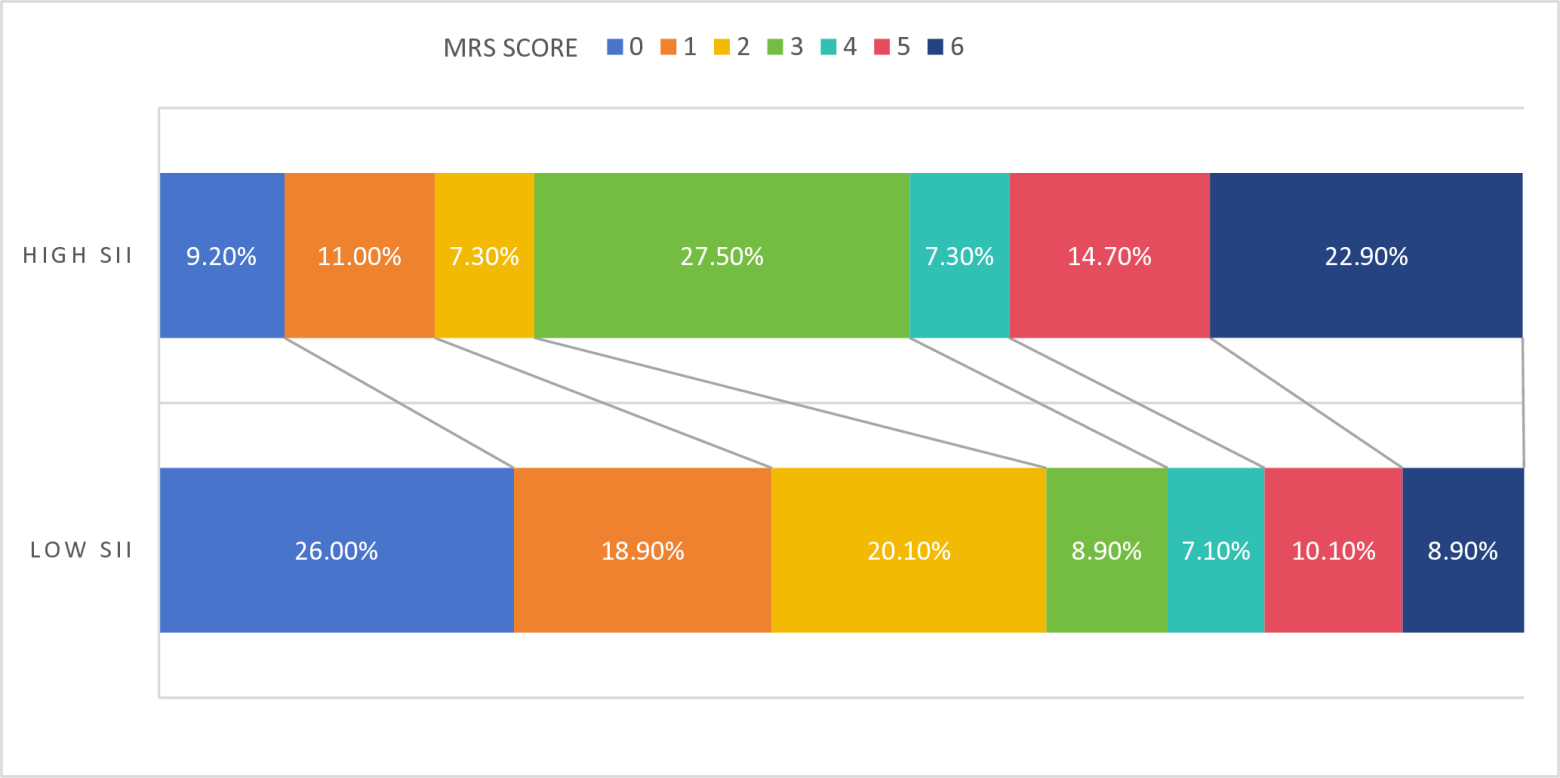
Association between the clinical outcome and high SII (the levels of SII > 652.73) and low SII. SII, systemic immune-inflammation index; mRS score, the modified Rankin Scale score.

Furthermore, the association between the clinical outcome and high SII (the levels of SII > 652.73) and low SII (the levels of SII < 652.73) was shown in Fig 3.

### Association of SII, NLR and PLR with brain edema

According to the SITS-MOST standard, brain edema was present in 61.9% (172/278) patients. CED-1, CED-2 and CED-3 were present in 98 (35.3%), 39 (14.0%), and 35 (12.6%), respectively. Compared to patients with non-malignant brain edema, patients with malignant brain edema were older [78 (71-83) vs. 74 (65-81), p =  0.044], had higher systolic blood pressure [164.20 ± 25.73 vs. 154.83 ± 24.10, p =  0.034], and higher levels of D-dimer, INR, FDP, FIB, and blood glucose [1273.00 (539.00-3163.00) vs. 514.00 (212.00,1512.00), p <  0.001; 1.05 (1.01-1.11) vs. 1.03 (0.97,1.08), p =  0.046; 15.07 (3.19-38.10) vs. 5.61 (1.99-17.86), p =  0.003; 4.08 ± 0.94 vs. 3.73 ± 0.87, p =  0.030; 8.15 (7.04-10.50) vs. 7.19 (6.08,9.09), p =  0.013]. They also had higher initial NIHSS and 24-hour NIHSS scores [16 (13–18) vs. 8 (5–14), p <  0.001; 17 (13–20) vs. 5 (2–11), p <  0.001], with less functional improvement or even deterioration after thrombolysis: NIHSS difference [0 (–3-2) vs. 2 (0–5), p < 0.001]. However, SII, NLR and PLR were not associated with the development of cerebral edema (Table 1).

The ASPECTS score on admission and the NIHSS score at 24 hours after admission were still independently associated with MCE ([OR], 0.490 [95% CI, 0.329-0.729], p < 0.001; [OR], 1.178 [95% CI, 1.086-1.279], p < 0.001) in binary logistic regression analysis model (Table 4).

**Table 4:** Binary logistic analyses for the prediction of malignant brain edema in acute ischemic stroke patients who underwent IVT.

Variables	OR	95%CI	P value
Age	0.986	0.941–1.033	0.555
SBP	1.011	0.993–1.028	0.230
24h NIHSS score	1.178	1.086–1.279	**<0.001**
NIHSS deference	1.025	0.931–1.128	0.615
ASPECTS score	0.490	0.329–0.729	**<0.001**
glucose	1.034	0.953–1.122	0.417
D-dimer	1.000	1.000–1.000	0.919
INR	0.516	0.004–70.767	0.792
FIB	1.600	0.995–2.574	0.053
FDP	1.001	0.993–1.009	0.817

SBP, systolic blood pressure; NIHSS score, the national institute of health stroke scale score; ASPECT score, alberta stroke program early CT score; INR, international normalized ratio; FIB, fibrinogen; FDP, fibrinogen degradation products.

## Discussion

The predictors of recent and long-term functional outcomes for AIS patients after IVT were uncertain. As inflammatory biomarkers, SII, NLR and PLR are easy to acquire, calculate and cost-effective. More importantly, they do not delay the time of thrombolysis, and could be monitored continuously. In this study, we revealed that the value of SII, PLR and NLR were associated with unfavorable outcomes at 3 months.

Numerous studies have demonstrated that the process of neuronal death involves pro-inflammatory cytokines. These cytokines can potentiate a complicated immune response to cerebral damage through immune cells such as lymphocytes, neutrophils, and monocytes [23]. Increased neutrophil concentration will promote the expression of matrix metalloproteinase-9 (MMP-9). Peripheral circulating neutrophils will release particles containing antibacterial enzymes and chemicals, which exacerbate brain injury. The inflammatory mediators, cytokines, adhesion molecules, and chemokines released by immune inflammatory cells worsen tissue damage. Besides, monocytes can also infiltrate into infarcted areas and aggravate brain injury. To the contrast, certain lymphocytes largely serve a protective role in AIS [13,24–26].

Studies by Fei Ma et al. indicated that increased baseline SII value was an independent risk factor for 3-month unfavorable outcomes in patients who underwent IVT, our study had drawn similar conclusions [16,17].

In our study, we revealed that SII, NLR and PLR were independent risk factors in the adjusted binary logistic regression analysis. Besides, SII had the best predictive power among the three indexes. This is attributed to the fact that NLR is predominantly associated with inflammation and can serve as an indicator for stroke-related pneumonia [27,28], which occurs in approximately 14%-27.8% of patients [29]. PLR is more focused on the aspects of hemostasis and thrombosis, while SII provides a comprehensive view of inflammation, hemostasis, immunity, and thrombosis throughout the body [30].

In addition, we indicated that MCE was an independent risk factor for 3-months unfavorable outcomes. MCE occurs in the first 1-3 days after stroke onset, clinical outcome exacerbated when severe edema develops [31,32], its diagnosis often based on clinical presentation, typical clinical course and neuroradiological findings, and lack of effective predictors [33,34]. Inflammation plays an important role in the formation of MCE and sICH, which were regarded as severe complications after IVT, and is closely related to the unfavorable outcomes of AIS. The SII value had been proved can independently and strongly predict 6-month unfavorable outcomes in patients with basal ganglia ICH and was positively correlated with the volume of cerebral hemorrhage [15]. Increased baseline SII was associated with early neurological deterioration with 24 hours after IVT [16]. For the first time, we investigated the relationship between SII, NLR and PLR and MCE for AIS patients who underwent IVT. But they were not significantly correlated with MCE in our study. The finding indicated the occurrence of malignant brain edema is not only associated with inflammatory responses but also with factors such as cerebral blood flow perfusion, blood-brain barrier disruption, and oxidative stress. The limited sample size in this study has also influenced the results. Besides, we did not analyze the dynamic changes with repeated measurements.

Moreover, our study of the relationship between SII, malignant brain edema and poor prognosis further highlighted the importance of systemic inflammatory responses in unfavorable outcomes. The high levels of inflammatory biomarkers hint the probability of complications other than brain edema, such as stroke-related pneumonia and ischemia reperfusion injury. On the one hand, our study found that the systolic blood pressure, ASPECTS scores, and NIHSS scores at 24 hours after admission significantly influenced the occurrence of malignant brain edema, this finding highlights the importance of timely recanalization and effectively managing blood pressure. On the other hand, in addition to timely vascular recanalization, monitoring blood pressure and treatment cerebral edema, our research underscores the criticality of proactive management of complications such as infections like stroke-related pneumonia. It provides some reference for the clinical treatment decisions concerning post-thrombolysis. Weather patients with elevated levels of SII, NLR or PLR before thrombolysis should receive more aggressive anti-infective treatment or blood pressure control therapy warrants further exploration.

Nonetheless, it’s a small, retrospective, single-center observational study. Further prospective studies with larger numbers of patients should be conducted to get the optimal full process management for IVT.

## Conclusions

Elevated SII, NLR and PLR are associated with unfavorable clinical outcomes. Clinicians should pay more attention to treating the complication of pulmonary infection, which may improve the prognosis of AIS patients.
