# Correction: Systemic immune-inflammation index is associated with clinical outcome of acute ischemic stroke patients after intravenous thrombolysis treatment

**DOI:** 10.1371/journal.pone.0332706

**Published:** 2025-09-16

**Authors:** Yuanfeng Zhou, Qian Yang, Zhangming Zhou, Xin Yang, Danni Zheng, Zhongchun He, Yizhou Liu, Tianzhu Xu, Ying Yin, Wenhui Wei, Chunli Si, Bozhi Zhang, Jianping Yu

There are errors in the author affiliations. The correct affiliations are as follows:

Yuanfeng Zhou^1,2^, Qian Yang^1,2^, Zhangming Zhou^3^, Xin Yang^1^, Danni Zheng^4^, Zhongchun He^2^, Yizhou Liu^2^, Tianzhu Xu^1^, Ying Yin^2^, Wenhui Wei^2^, Chunli Si^5^, Bozhi Zhang^1^, Jianping Yu^1,2^

1 School of Clinical Medicine, Chengdu Medical College, 2 Department of Neurology, The First Affiliated Hospital of Chengdu Medical College, Chengdu, Sichuan, China, 3 Department of Neurosurgery, Dujiangyan Medical Center, Chengdu, Sichuan, China, 4 Biomedical Informatics and Digital Health, School of Medical Sciences, University of Sydney, Sydney, Australia, 5 Department of Medical Administration, The First Affiliated Hospital of Chengdu Medical College, Chengdu, Sichuan, China.
